# Postacute Care Services Use and Outcomes Among Traditional Medicare and Medicare Advantage Beneficiaries

**DOI:** 10.1001/jamahealthforum.2023.2517

**Published:** 2023-08-18

**Authors:** Emma M. Achola, David G. Stevenson, Laura M. Keohane

**Affiliations:** 1 Department of Health Policy, Vanderbilt University Medical Center, Nashville, Tennessee; 2 Geriatric Research, Education, and Clinical Center, Veterans Affairs Tennessee Valley Healthcare System, Nashville

## Abstract

**Importance:**

Better evidence is needed on whether Medicare Advantage (MA) plans can control the use of postacute care services while achieving excellent outcomes.

**Objective:**

To compare self-reported use of postacute care services and outcomes among traditional Medicare (TM) beneficiaries and MA enrollees.

**Design, Setting, and Participants:**

This cohort study used data from the National Health and Aging Trends Study (NHATS) with linked Medicare enrollment data from 2015 to 2017. Participants were community-dwelling MA or TM beneficiaries 70 years and older; those with dual Medicare and Medicaid eligibility were also identified. Analyses were conducted from May 2022 to February 2023 and were weighted to account for the complex survey design.

**Exposures:**

Enrollment in MA and dual eligibility for Medicare and Medicaid.

**Main Outcomes and Measures:**

Postacute care service use including site of use, duration, primary indication, and whether participants met their goals or experienced improved functional status during or after services.

**Results:**

Included in the analysis were 2357 Medicare beneficiaries who used postacute care. Of these beneficiaries, 815 (32.6%; 62.0% were females [weighted percentages]) had MA and 1542 (67.4%; 59.5% were females [weighted percentages]) had TM. Enrollees in MA reported using postacute care services across all NHATS survey rounds: between 16.2% (95% CI, 14.3%-18.4%) and 17.7% (95% CI, 15.4%-20.4%) of MA enrollees reported using postacute care services each round, vs 22.4% (95% CI, 20.9%-24.1%) to 24.1% (95% CI, 21.8%-26.6%) of TM beneficiaries (*P* for all rounds <.002). Enrollees in MA reported less functional improvement during postacute care use (63.1% [95% CI, 59.2%-66.8%] vs 71.7% [95% CI, 68.9%-74.3%], *P* < .001). Among beneficiaries who ended postacute service use, fewer MA enrollees than TM enrollees reported that they met their goals (70.5% [95% CI, 65.1%-75.3%] vs 76.2% [95% CI, 73.1%-79.1%]; *P* = .053) or had improved functional status (43.9% [95% CI, 38.9%-49.1%] vs 46.0% [95% CI, 42.5%-49.5%]; *P* = .42), but differences were not statistically significant. Differences in postacute care use and functional improvement were not statistically significant between MA and TM enrollees with dual eligibility.

**Conclusions and relevance:**

In this cohort study of Medicare beneficiaries, we found that MA enrollees overall used less postacute care services than their TM counterparts. Among users of postacute care services, MA enrollees reported less favorable outcomes compared with TM enrollees. These findings highlight the importance of assessing patient-reported outcomes, especially as MA and other payment models seek to reduce inefficient use of postacute care services.

## Key Points

**Question:**

Are there differences in the use of postacute care services and outcomes between Medicare Advantage (MA) and traditional Medicare beneficiaries?

**Findings:**

In this cohort study of 2357 Medicare beneficiaries who used postacute care services, MA enrollees reported less use of postacute care services and shorter duration of services vs traditional Medicare beneficiaries. Fewer MA enrollees reported functional improvement while using postacute care.

**Meaning:**

Findings of this study suggest the importance of understanding differences in postacute care service use and outcomes by enrollment status; self-reported outcomes are especially important as MA and other payment models seek to reduce use of postacute care services.

## Teaser

This cohort study examines differences in the use and self-reported outcomes of postacute care services between traditional Medicare and Medicare Advantage beneficiaries.

## Introduction

After a hospitalization, Medicare beneficiaries often receive postacute care services from skilled nursing facilities (SNFs), inpatient rehabilitation facilities (IRFs), long-term care hospitals, outpatient clinics, or home health agencies. Medicare also covers postacute care without a hospital stay through noninstitutional facilities, such as home health agencies and outpatient rehabilitation facilities. Traditional Medicare spending on postacute care totaled $57.9 billion in 2020,^1^ prompting questions about how Medicare can expand payment models that promote more efficient use of postacute care services.

Traditional Medicare (TM) and Medicare Advantage (MA) have different incentives that affect postacute care use. Traditional Medicare’s largely fee-for-service reimbursement system potentially encourages overuse of postacute care services. In contrast, capitated rates paid to MA plans may incentivize plans to steer patients to less expensive settings, limit service duration,^2^ or refuse prior authorization for postacute care.^3^ Understanding differences in postacute care services between these 2 populations is important as MA enrollment continues to grow; MA enrollment is estimated to reach approximately 61% by 2032.^4^ This growth includes increasing numbers of MA enrollees with low incomes who have dual Medicare and Medicaid eligibility,^1,5^ have high rates of postacute care use,^6^ and may be more adversely affected by efforts to limit use of postacute services.

Prior studies comparing postacute care use and outcomes between TM and MA beneficiaries have largely found that MA beneficiaries use fewer postacute care services^3,7,8,9^ and generally have favorable outcomes even with differential use of these services.^3,7,8^ Among beneficiaries who were hospitalized for lower extremity joint replacement, stroke, or heart failure, MA enrollees compared with TM beneficiaries were discharged to IRFs less often, experienced shorter lengths of stay in an SNF,^3,8^ and were more likely to be discharged home without any institutional or home health care.^3^ These findings align with other work demonstrating less home health and SNF use among patients with MA vs TM.^10^ Furthermore, MA enrollees with hip fractures experienced shorter lengths of stay in SNFs and fewer minutes of rehabilitation compared with TM beneficiaries.^7^

To our knowledge, less postacute care use among MA enrollees has not had measurable associations with unfavorable outcomes in studies to date. Compared with TM beneficiaries, MA enrollees were more likely to experience a successful discharge to community^7^ and were more likely to remain in the community postdischarge.^8^ Enrollees in MA also were less likely to be readmitted postdischarge, with no mortality differences between the 2 groups.^8^ However, these studies exclusively relied on administrative data sources and did not capture patients’ experiences with postacute care. Examining self-reported patient outcomes is key to ensuring that the MA program adequately meets beneficiaries’ needs, particularly since there is evidence that MA enrollees are treated at lower-quality SNFs.^11^

Herein we examined differences in self-reported use of postacute care (includes services provided with and without prior hospitalization) and outcomes among community-dwelling beneficiaries enrolled in TM and MA. Understanding how beneficiaries perceive the implications of postacute care services for functional outcomes is important, particularly since other studies have largely used only administrative data sources. We also examined differences in postacute care use and outcomes for beneficiaries with dual eligibility for Medicare and Medicaid to understand if MA enrollment was associated with different postacute care patterns for this population.

## Methods

### Data Sources

We used data from the National Health and Aging Trends Study (NHATS) for self-reported measures of postacute care use and outcomes. The NHATS is a nationally representative cohort of Medicare beneficiaries aged 65 years and older. Data on MA and dual-eligibility enrollment were available from linked Master Beneficiary Summary File records. We focused on survey rounds 5 through 7 (corresponding with interviews conducted between 2015 and 2017) because NHATS began including questions about postacute care services in survey round 5 and Medicare enrollment data were available through round 7. The Johns Hopkins Institutional Review Board approved the NHATS protocol; all participants provided informed consent. This study was approved by the Vanderbilt Institutional Review Board with a waiver of consent to examine linked Medicare records. This cohort study followed the Strengthening the Reporting of Observational Studies in Epidemiology (STROBE) reporting guideline (eMethods in Supplement 1).

### Study Population

Study participants were individuals aged 70 years or older who completed all postacute care survey questions and had linked Medicare enrollment data. Since we examined trends over time, we followed NHATS technical guidance to ensure that the sample was representative of the national Medicare population for this age group.^12^ Participants were community dwelling, meaning they reported living in the community or a residential facility other than a nursing home; eFigure 2 in Supplement 1 details study population selection criteria.

Participants were categorized as having MA if they were enrolled in MA for at least 1 month during the calendar year when the survey was fielded; otherwise, participants were categorized as having TM. Participants with at least 1 month of partial or full Medicaid enrollment during the calendar year when the study was fielded were categorized as having dual eligibility for Medicare and Medicaid.

We performed analyses for subpopulations more likely to use postacute care: dual-eligible beneficiaries and individuals who self-reported a hospital stay, fall, or hip or knee surgery in the past year, or had a history of arthritis or osteoporosis. By focusing on populations who experienced similar health events or conditions in the past year, we aimed to minimize any unobserved differences in health or functional status between MA and TM enrollees.

### Main Study Measures

Participants completed a validated set of questions about whether they received postacute care services in the past year, including questions about the duration of use.^13^ Among participants with postacute care use, follow-up questions ascertained whether these services were delivered in an inpatient setting (ie, an overnight hospital, nursing home, or rehabilitation facility), outpatient setting (ie, an outpatient center, clinic, or physician’s or therapist’s office), or at home. The inpatient postacute care measure did not differentiate between SNFs or IRFs. After assessing whether participants’ postacute care use followed surgery, the survey asked participants about the medical indication for surgery. If participants did not have surgery, the survey instead captured the main medical condition for which they received postacute care. eTable 3 in Supplement 1 describes NHATS survey questions on postacute care services use.

Among participants with postacute care use, we examined the following self-reported outcomes: whether the participant’s physical functioning improved while receiving postacute care or after postacute care services ended and whether they had met their care goals when services ended. If the use of postacute care was ongoing, participant responses were not included in these latter 2 categories.

Finally, we assessed the following characteristics: age, gender, self-reported race and ethnicity, education level, marital status, household size, self-reported health status, frailty indicators,^14,15^ cognitive status,^16^ and any self-reported hospital stays, falls, hip or knee surgeries, or osteoporosis or arthritis diagnoses. We included data on race and ethnicity due to prior evidence that MA participation has increased substantially over time among Black and Hispanic beneficiaries.^17^ Race and ethnicity categories were Black, White, Hispanic, and other or not reported (including American Indian, Asian, Native Hawaiian, Pacific Islander, or other race or ethnicity specified by respondent).

### Statistical Analysis

All analyses were weighted to account for the complex survey design. We used Pearson χ^2^ tests for comparisons of MA and TM participants, reporting frequencies (%) and corresponding 95% CIs. We first identified the proportion of study participants who used postacute care in each NHATS survey round. Among beneficiaries who used postacute care services, we pooled our analysis across survey rounds 5 through 7 by taking participants’ first reported instance of postacute care service use. We examined whether characteristics of participants who used postacute care services differed by MA enrollment in the overall population and in the dual-eligible population. For the previously described measures on use of postacute care services and outcomes, we compared results for the MA and TM beneficiaries. We followed the same analytic approach for analyses of postacute care use and outcomes among dual-eligible beneficiaries and other subgroups.

Statistical analyses were conducted from May 2022 to February 2023, using Stata/MP, version 14.2 (Stata Corp). Results with a 2-sided *P* < .05 were considered significant.

## Results

### Population Characteristics

**Figure 1.**
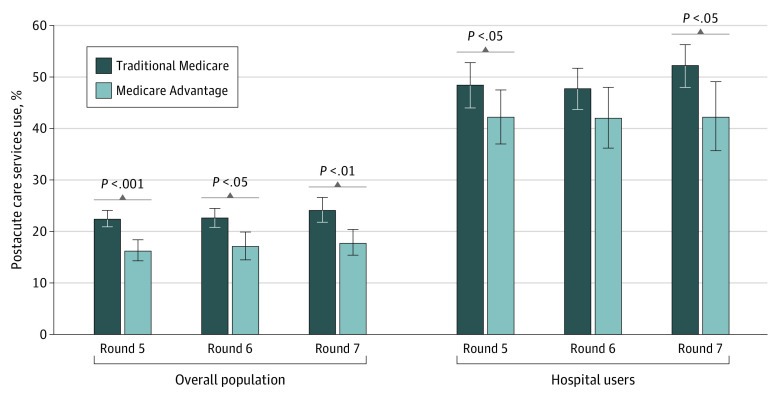
Use of Postacute Care Services by National Health and Aging Trends Study Survey Round and Managed Care Population, 2015-2017 Percentages were weighted to account for the complex survey design. Hospital users are those who reported at least 1 hospital stay in the past 12 months. Between-group comparisons based on Pearson χ^2^ tests. Error bars indicated 95% CIs.

Of 16 946 community-dwelling participants eligible for inclusion in the analysis, 2357 participants self-reported postacute care use in rounds 5 through 7 of the NHATS survey. Of these participants, 1542 (67.4%) had TM (59.5% were female and 40.5% were male [weighted percentages]) and 815 (32.6%) had MA (62.0% were females and 38.0% were males [weighted percentages]). Among those with TM, 5.8% were Black, 4.2% were Hispanic, and 83.6% were White individuals and 6.4% were individuals with other or not reported race and ethnicity. Among those with MA, 8.7% were Black, 7.5% were Hispanic, and 78.1% were White individuals and 5.6% were individuals with other or not reported race and ethnicity. Enrollees in MA reported less use of postacute care services than TM beneficiaries across all survey rounds (Figure 1). Between 16.2% (95% CI, 14.3%-18.4%) and 17.7% (95% CI, 15.4%-20.4%) of MA enrollees reported using postacute care services each round compared with 22.4% (95% CI, 20.9%-24.1%) to 24.1% (95% CI, 21.8%-26.6%) of TM beneficiaries (*P* < .002 for all rounds). Among participants who experienced a hospitalization, MA beneficiaries used fewer postacute care services than TM beneficiaries, with significant differences in round 5 (42.2% [95% CI, 37.0%-47.5%] vs 48.4% [95% CI, 44.0%-52.8%]; *P* = .04) and round 7 (42.2% [95% CI, 35.7%-49.1%] vs 52.2% [95% CI, 48.0%-56.3%]; *P* = .03). Dual-eligible MA beneficiaries also reported less use of postacute services in each round compared with dual-eligible TM beneficiaries, but the difference was not statistically significant (eFigure 1 in Supplement 1).

We observed few significant differences in the characteristics of participants who used postacute care by MA enrollment (Table 1). More participants with postacute care use who had MA were dual eligible for Medicare and Medicaid (17.8% [95% CI, 14.1%-22.3%]) compared with those with postacute care use who had TM (11.1% [95% CI, 9.2%-13.3%]; *P* = .002). Compared with those with TM, more MA enrollees with postacute care use had less than a high school education (47.9% [95% CI, 43.6%-52.2%] vs 38.3% [95% CI, 34.2%-42.5%]; *P* = .004) and were non-Hispanic Black (8.7% [95% CI, 6.7%-11.2%] vs 5.8% [95% CI, 4.9%-6.8%]) or Hispanic individuals (7.5% [95% CI, 5.4%-10.5%] vs 4.2% [95% CI, 3.2%-5.7%]; *P* = .02). A higher proportion of MA participants reported being in fair (22.5% [95% CI, 18.7%-26.9%] vs 21.6% [95% CI, 18.9%-24.6%]) or poor health (11.2% [95% CI, 8.5%-14.6%] vs 7.4% [95% CI, 5.9%-9.2%]; *P* = .03). The distribution of dementia status varied, with more MA enrollees compared with TM beneficiaries who used postacute care services having probable dementia (15.1% [95% CI, 11.9%-18.9%] vs 11.4% [95% CI, 9.7%-13.5%]) and fewer MA enrollees having possible dementia (6.0% [95% CI, 4.4%-8.1%) vs 8.7% [ 95% CI, 7.1%-10.6%]; *P* = .03). Half of Medicare enrollees who used postacute care services reported a hospital stay in the previous year regardless of enrollment in TM (49.4% [95% CI, 46.3%-52.5%]) or MA (51.7% [95% CI, 48.1%-55.3%]; *P* = .29). Among dual-eligible enrollees who used postacute care services, we observed no significant differences in most beneficiary characteristics (Table 1), although fewer MA enrollees with dual eligibility who used postacute care services had osteoporosis or arthritis than their TM counterparts (71.1% [95% CI, 61.2%-79.4%] vs 82.8% [95% CI, 75.1%-88.4%]; *P* = .04).

**Table 1.** Characteristics of Medicare Beneficiaries With Postacute Care Use, Stratified by Managed Care Participation and Dual Eligibility for Medicare and Medicaid, 2015 to 2017^a^

Characteristic	Weighted overall study population, % (95% CI)^b^		Weighted dual-eligible population, % (95% CI)^b^	
	With TM	With MA	With TM	With MA
Weighted population, No. (%)	8 003 310 (67.4)	3 875 021 (32.6)	886 318 (56.2)	691 612 (43.8)
Age, y				
70-74	36.8 (33.5-40.2)	37.6 (33.6-41.7)	37.1 (27.9-47.2)	33.4 (23.0-45.7)
75-79	24.4 (21.7-27.3)	24.9 (21.2-28.9)	19.3 (13.3-27.1)	30.3 (22.1-39.9)
≥80	38.8 (36.3-41.3)	37.6 (33.9-41.4)	43.6 (34.9-52.8)	36.3 (26.6-47.3)
Gender				
Female	59.5 (56.4-62.5)	62.0 (58.4-65.4)	65.7 (57.8-72.9)	68.6 (58.5-77.1)
Male	40.5 (37.5-43.6)	38.0 (34.6-41.6)	34.3 (27.1-42.2)	31.4 (22.9-41.5)
Race and ethnicity				
Black	5.8 (4.9-6.8)	8.7 (6.7-11.2)	13.3 (9.3-18.6)	18.5 (12.6-26.5)
Hispanic	4.2 (3.2-5.7)	7.5 (5.4-10.5)	17.7 (11.7-25.9)	22.8 (14.3-34.5)
White	83.6 (81.3-85.7)	78.1 (73.1-82.4)	50.4 (40.1-60.7)	47.7 (34.9-60.8)
Other or not reported^c^^,^^d^	6.4 (4.9-8.3)	5.6 (3.4-9.3)	18.6 (11.3-29.1)	10.9 (5.7-20.0)
Education				
High school or less	38.3 (34.2-42.5)	47.9 (43.6-52.2)	69.4 (61.4-76.4)	71.4 (61.3-79.8)
Some college	26.6 (23.9-29.6)	26.6 (22.9-30.8)	NA	NA
College degree or higher	32.2 (28.5-36.2)	24.0 (19.7-28.9)	NA	NA
Not reported	2.9 (1.9-4.4)	1.5 (0.7-3.0)	NA	NA
Some college, college degree or higher, or not reported	NA	NA	30.6 (23.6-38.6)^e^	28.6 (20.2-38.7)^e^
Dual eligibility				
Medicare only	88.9 (86.7-90.8)	82.2 (77.7-85.9)	NA	NA
Dual eligible	11.1 (9.2-13.3)	17.8 (14.1-22.3)	NA	NA
Marital status				
Married or living with a partner	54.7 (51.7-57.5)	51.4 (47.7-55.1)	21.5 (14.9-30.1)	26.1 (18.1-36.2)
Previously married	42.2 (39.5-44.9)	46.3 (42.9-49.8)	NA	NA
Never married	3.2 (2.3-4.4)	2.3 (1.5-3.5)	NA	NA
Previously married or never married	NA	NA	78.5 (69.9-85.1)^e^	73.9 (63.8-81.9)^e^
No. living in household				
Lives alone	33.7 (30.8-36.8)	31.1 (27.8-34.6)	51.9 (42.8-60.9)	41.6 (32.1-51.7)
2 People	51.9 (49.3-54.5)	50.9 (46.3-55.5)	25.6 (20.1-32.0)	31.1 (22.6-41.3)
≥3 People	14.4 (12.3-16.6)	17.9 (14.4-22.0)	22.5 (16.1-30.5)	27.3 (20.1-35.8)
Self-reported health status				
Excellent or very good	37.1 (34.2-40.1)	31.7 (28.0-35.7)	14.2 (9.9-19.9)	13.8 (8.1-22.6)
Good	34.0 (31.4-36.6)	34.5 (30.8-38.5)	25.5 (17.7-35.2)	27.1 (19.2-36.8)
Fair	21.6 (18.9-24.6)	22.5 (18.7-26.9)	41.8 (32.9-51.3)	36.6 (26.4-48.3)
Poor or not reported^f^	7.4 (5.9-9.2)	11.2 (8.5-14.6)	18.5 (11.9-27.7)	22.4 (13.4-35.0)
Hospital stay in past year^f^	49.4 (46.3-52.5)	51.7 (48.1-55.3)	62.8 (53.2-71.5)	65.2 (55.5-73.9)
Fall in past year	49.8 (46.5-53.1)	46.1 (42.7-49.6)	70.6 (60.9-78.7)	58.3 (48.5-67.4)
Hip or knee surgery in past year	13.0 (11.1-15.1)	12.7 (10.7-15.0)	7.6 (4.3-13.0)	7.4 (3.9-13.7)
History of osteoporosis or arthritis	80.3 (77.8-82.6)	76.7 (73.0-80.1)	82.8 (75.1-88.4)	71.1 (61.2-79.4)
Dementia status^g^				
Probable dementia	11.4 (9.7-13.5)	15.1 (11.9-18.9)	26.1 (17.2-37.7)	33.6 (23.4-45.6)
Possible dementia	8.7 (7.1-10.6)	6.0 (4.4-8.1)	20.4 (13.6-29.4)	8.4 (4.6-14.8)
Frailty status^g^				
Pre-frail	46.3 (43.4-49.3)	48.1 (44.1-52.1)	40.7 (32.6-49.3)	39.7 (30.2-49.9)
Frail	25.3 (22.6-28.1)	26.9 (23.5-30.6)	50.1 (41.0-59.1)	49.5 (38.5-60.5)

Abbreviations: MA, Medicare Advantage; NA, not applicable; TM, traditional Medicare.

^a^ Data source: National Health and Aging Trends Survey data linked to Medicare enrollment data, years 2015 to 2017.

^b^ Percentages were weighted to account for complex survey design. In the overall population, the unweighted number of participants with TM or MA was 1542 and 815, respectively. In the dual-eligible population, the unweighted number of participants with TM or MA was 207 and 168, respectively.

^c^ Between 26 and 50 participants in the overall population were missing information on this measure.

^d^ Other category includes multiracial, not reported, or American Indian, Asian, Native Hawaiian, Pacific Islander, or other race or ethnicity specified by respondents.

^e^ Due to data use agreement cell size restrictions, some measures combine categories when reporting.

^f^ Fewer than 11 participants in the overall population were missing information on this measure.

^g^ Between 11 and 25 participants in the overall population were missing information on this measure.

### Characteristics of Postacute Care Use

Among Medicare beneficiaries who used postacute care services, MA enrollees reported a shorter duration of postacute care use compared with TM beneficiaries (Table 2). Roughly one-third (30.3% [95% CI, 26.3%-34.7%]) of MA enrollees reported receiving postacute services for 4 weeks or less compared with 24.3% ([95% CI, 21.9%-26.9%; *P* = .005]) of TM enrollees. We observed no significant differences between the 2 populations in site of postacute care services or the condition or procedure that prompted postacute care use. Regardless of MA enrollment, nearly half of participants (TM beneficiaries: 46.0% [95% CI, 43.1%-48.9%]; MA enrollees: 45.2% [95% CI, 41.0%-49.4%]) reported receiving postacute care services solely in an outpatient setting, and roughly one-third (TM beneficiaries: 33.2% [95% CI, 30.3%-36.3%]; MA enrollees: 32.1% [95 CI, 28.4%-36.0%]) reported postacute care use after surgery.

**Table 2.** Self-Reported Use and Outcome Measures Among Postacute Care Users by Managed Care Enrollment and Dual Eligibility for Medicare and Medicaid^a^

	Weighted overall study population, % (95% CI)^b^		Weighted dual-eligible population, % (95% CI)^b^	
	With TM	With MA	With TM	With MA
Duration of postacute care use^c^				
≤2 wk	13.9 (11.8-16.2)^c^	17.6 (14.1-21.8)^c^	10.3 (6.2-16.8)	18.6 (11.4-28.9)
3-4 wk	10.4 (8.6-12.7)	12.7 (10.1-15.8)	10.3 (5.6-18.3)	12.4 (7.0-21.0)
1-3 mo	60.0 (56.9-63.0)	60.0 (55.3-64.5)	58.4 (50.3-66.0)	56.9 (47.7-65.7)
4-5 mo	8.5 (6.8-10.6)	5.8 (4.4-7.5)	NA	NA
≥6 mo	7.2 (5.9-8.8)	3.9 (2.7-5.6)	NA	NA
≥4 mo	NA	NA	21.0 (15.9-27.2)	12.1 (7.2-19.6)
Site of postacute care				
Inpatient only	8.2 (6.8-9.9)	6.2 (4.5-8.4)	13.0 (7.7-21.1)	8.4 (4.3-16.0)
Outpatient only	46.0 (43.1-48.9)	45.2 (41.0-49.4)	27.8 (19.4-38.2)	24.9 (17.8-33.8)
Home only	12.6 (10.9-14.6)	14.3 (11.6-17.4)	22.8 (16.3-31.1)	29.1 (20.8-39.0)
≥2 Sites, 1 was inpatient	25.3 (22.9-27.9)	26.8 (23.7-30.2)	32.1 (24.9-40.3)	30.8 (23.2-39.6)
≥2 Sites, none was inpatient, somewhere else, or not reported	7.9 (6.2-9.9)	7.6 (5.8-9.8)	4.2 (2.3-7.6)	6.8 (3.3-13.3)
Primary reason for receiving postacute care				
Surgical				
Fracture, sprain, or injury	5.5 (4.2-7.1)	5.4 (3.9-7.4)	NA	NA
Fracture, sprain, or injury or another musculoskeletal condition	NA	NA	7.0 (2.6-17.2)	9.7 (5.5-16.5)
Hip, knee, or other joint replacement	11.4 (9.9-13.2)	12.3 (10.2-14.7)	6.4 (3.5-11.1)	8.6 (4.6-15.4)
Another musculoskeletal condition	5.8 (4.5-7.5)	5.2 (3.6-7.4)	NA	NA
Other^d^	10.4 (8.6-12.7)	9.3 (7.2-11.8)	13.5 (8.7-20.4)	11.4 (6.2-20.3)
Nonsurgical				
Fracture, sprain, or injury	14.7 (12.4-17.4)	14.1 (11.4-17.4)	14.0 (8.2-23.0)	14.0 (8.8-21.5)
Hip, knee, or other joint replacement, or another musculoskeletal condition	28.1 (25.5-30.8)	28.4 (24.7-32.3)	22.9 (16.7-30.6)	22.7 (15.4-32.0)
Stroke or TIA	2.7 (2.1-3.6)	4.6 (3.2-6.5)	5.8 (3.6-9.2)	6.5 (3.3-12.4)
Other^e^	21.3 (18.9-23.9)	20.9 (17.7-24.5)	30.4 (22.3-40.0)	27.2 (18.2-38.5)

Abbreviations: MA, Medicare Advantage; NA, not applicable; TM, traditional Medicare; TIA, transient ischemic attack.

^a^ Data source: National Health and Aging Trends Survey data linked to Medicare enrollment data, years 2015 to 2017.

^b^ Percentages were weighted to account for complex survey design. In the overall population, the unweighted number of participants with TM or MA was 1542 and 815, respectively. In the dual-eligible population, the unweighted number of participants with TM or MA was 207 and 168, respectively.

^c^ Overall duration, MA vs TM, *P* < .005 based on Pearson χ^2^ tests.

^d^ The other category includes stroke, TIA, heart attack, heart condition or vascular disease, breathing condition, neurological condition, cancer, no medical condition, or another condition.

^e^ The other category includes heart attack, heart condition or vascular disease, breathing condition, neurological condition, cancer, another condition, or no medical condition.

We found no statistically significant differences by enrollment status in postacute care service indication, duration, or location among dual-eligible beneficiaries (Table 2). Using only home-based services was common among dual-eligible MA (29.1% [95% CI, 20.8%-39.0%]) and TM enrollees (22.8% [95% CI, 16.3%-31.1%]; *P* = .53).

### Outcomes of Postacute Care Use

**Figure 2.**
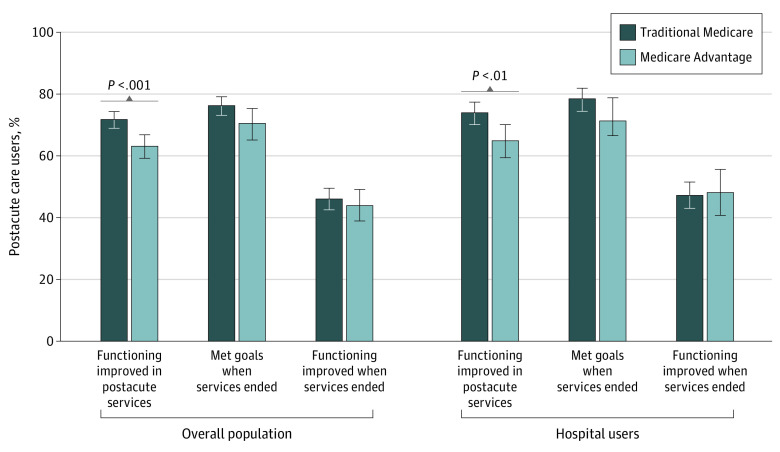
Self-Reported Outcomes by Managed Care Enrollment for Postacute Care Users Percentages were weighted to account for the complex survey design. Between-group comparisons based on Pearson χ^2^ tests. Participants reporting current use of postacute care were not included in the categories of *Met goals when services ended* or *Functioning improved when services ended*. Hospital users are those who reported at least 1 hospital stay in the past 12 months. Error bars indicated 95% CIs.

 Fewer MA enrollees reported improved functioning while receiving postacute care than TM beneficiaries among the overall population (Figure 2; 63.1% [95% CI, 59.2%-66.8%] and 71.7% [95% CI, 68.9%-74.3%]; *P* < .001) and among participants with a hospital stay (64.9% [95% CI, 59.4%-70.1%] and 73.9% [95% CI, 70.1%-77.4%]; *P* = .006). Overall, among beneficiaries who no longer used postacute services, we observed no statistically significant differences in self-reported functional improvement at the end of care (43.9% [95% CI, 38.9%-49.1%] of MA enrollees and 46.0% [95% CI, 42.5%-49.5%] of TM enrollees; *P* = .42) or in whether beneficiaries had met their goals (70.5% [95% CI, 65.1%-75.3%] of MA enrollees and 76.2% [95% CI, 73.1%-79.1%] of TM enrollees; *P* = .053).

**Figure 3.**
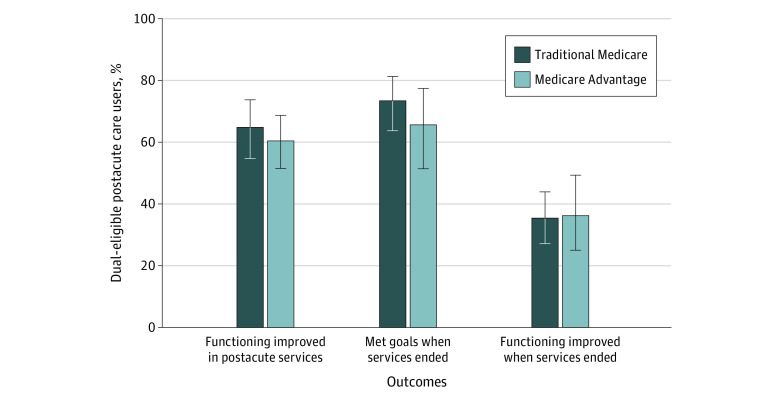
Self-Reported Outcomes by Managed Care Enrollment for Dual-Eligible Postacute Care Users Percentages were weighted to account for the complex survey design. Between-group comparisons based on Pearson χ^2^ test; no statistical differences found. Error bars indicated 95% CIs.

These findings were consistent, but not statistically significant, for the dual-eligible population (Figure 3). Fewer dual-eligible MA enrollees reported improved functioning while receiving postacute care (60.4% [95% CI, 51.5%-68.6%]) relative to dual-eligible TM beneficiaries (64.8% [95% CI, 54.7%-73.7%], *P* = .49). Fewer dual-eligible MA enrollees also reported that they met their goals compared with dual-eligible TM beneficiaries (65.6% [95% CI, 51.4%-77.4%] vs 73.4% [95% CI, 63.7%-81.3%], *P* = .34).

Subgroup analyses largely followed trends that were similar to those of the overall study population. Among beneficiaries who had a fall, hip or knee surgery, or arthritis or osteoporosis, MA enrollees generally reported less postacute care use than TM beneficiaries (eTable 1 in Supplement 1). Across all subgroups, fewer MA enrollees reported functional improvement while receiving postacute care services. For example, 76.0% (95% CI, 62.8%-85.7%) of MA enrollees with hip or knee surgery in the past year reported functional improvement vs 88.3% (95% CI, 81.1%-93.0%; *P* = .04) of TM enrollees (eTable 2 in Supplement 1).

## Discussion

In this cohort study of older community-dwelling Medicare beneficiaries, we found that fewer MA enrollees reported using postacute care services than TM beneficiaries. Among participants with postacute care use, MA enrollees had shorter duration of services, but the services did not differ from those used by TM beneficiaries in terms of location of care or reason for using these services. Enrollees in MA who used postacute care services reported less functional improvement while receiving these services.

Among participants with conditions that may make them more likely to use postacute care services (ie, participants with a self-reported hospitalization, fall, hip or knee surgery in the past year, or arthritis or osteoporosis), MA enrollees often reported less use of postacute care and less functional improvement while using these services compared with TM beneficiaries. Similar patterns were observed among dual-eligible beneficiaries, although differences between MA and TM dual-eligible enrollees were not statistically significant.

Results of the present study align with those of previous studies^3,7,8,9^ documenting that MA enrollees use fewer postacute care services than TM enrollees. Despite this differential use of services, previous studies based on administrative data have found that MA enrollees have better outcomes for some measures, such as readmissions and successful discharge to the community.^3,7,8^ The present study adds to the literature by using self-reported outcomes to capture self-perceived functional improvement among Medicare beneficiaries who used postacute care services and examining a broader range of rehabilitative services, such as outpatient clinics. Unlike prior studies, we found that MA enrollees who used postacute care services reported less favorable outcomes on some measures than TM beneficiaries.

Differences in perceptions of improvement may be related to MA utilization management practices. Enrollees in MA are more likely to use lower-quality SNFs compared with TM beneficiaries,^11^ perhaps due to practitioners available in MA plan networks. By requiring prior authorization for postacute care services, plans may delay patients’ receipt of these services or end such services before the beneficiary is ready,^2^ which could be associated with higher levels of dissatisfaction with care.

Findings of the present study have implications for Medicare’s value-based payment initiatives. Based on the evidence that postacute care spending contributes to variation in TM spending,^18^ several Medicare payment models in addition to MA have targeted reducing unnecessary use of postacute care services. According to evaluations of administrative data, the Medicare Shared Savings Program^19^ and mandatory bundled payments^20^ achieved savings by decreasing postacute care use without adverse outcomes. However, findings of the present study suggest that self-reported data from Medicare beneficiaries may introduce important evidence about potential declines in patient satisfaction that should be investigated as Medicare seeks to expand payment models that promote more efficient use of postacute care services.

### Limitations

As with all studies comparing MA and TM enrollees, differences in outcomes may reflect unobservable differences in health between these populations. Due to the favorable selection of healthier enrollees into the MA program,^21,22,23^ MA enrollees may be less likely to use postacute care than TM beneficiaries, which may partly explain our findings. However, this potential bias may be flipped when examining outcomes among participants who have postacute care use. Medicare Advantage plans can limit access to postacute care through utilization management practices, such as prior authorization. Accordingly, MA postacute care users may be in worse health than TM postacute care users. However, we found few significant differences by managed care enrollment across multiple characteristics of postacute care users. We also performed sensitivity analyses for subpopulations who experienced similar health events or had similar conditions and found comparable results for service use and functional improvement. Furthermore, self-reported outcomes, such as attaining functional improvement and meeting goals, should be less reflective of health status. In other words, beneficiaries should be able to expect satisfaction with care regardless of their level of acuity.

This study has several other limitations. First, small sample sizes in some subpopulations limited our ability to perform certain analyses and may have contributed to the lack of statistical significance for some findings. Studies using administrative data to compare TM and MA participants can more precisely estimate differences in postacute care use but lack self-reported data to understand beneficiary perspective. Second, we had limited information on when postacute care services were received relative to enrollment in MA. We could ascertain only that survey participants were enrolled in MA during the year the survey was fielded. Third, we excluded individuals residing in nursing homes, a particularly frail population with high rates of SNF use. Fourth, we did not have geographic data to account for regional variation in MA penetration or postacute care use. Fifth, despite knowing the condition that prompted participants’ postacute care use, we did not have more precise measures of participants’ level of functioning when initiating postacute care services. Participants with higher baseline functioning may have had less room to improve. Sixth, our analysis excluded postacute care users who died prior to survey completion, and we did not account for potential differences in mortality between TM and MA enrollees.

## Conclusions

In this cohort study of postacute care services use among TM and MA beneficiaries, we believe that the use of self-reported outcomes adds important evidence since patient-reported outcomes are needed to improve care delivery processes. As enrollment in MA continues to grow, understanding differences in the use of services and outcomes by enrollment status is important, particularly if differential service use is associated with perceived differences in care for MA enrollees. We found that MA enrollment was associated with less improvement in self-reported outcomes. These findings highlight the importance of self-reported outcomes, especially as MA and other payment models seek to reduce use of postacute care services.

## Notes

Supplement 1. **eMethods.** STROBE Compliance **eFigure 1.** Dual-Eligible Postacute Care Service Use by NHATS Survey Round and Managed Care Enrollment, 2015-2017 **eTable 1.** Self-Reported Use of Postacute Care by NHATS Survey Round and Managed Care Enrollment for Subgroups **eTable 2.** Self-Reported Outcomes by Managed Care Enrollment for Subgroups **eTable 3.** NHATS Survey Questions Related to Postacute Care **eFigure 2.** CONSORT Diagram for Study Population

Supplement 2.
Data Sharing Statement
