# Supplementary material for: Postacute Care Services Use and Outcomes Among Traditional Medicare and Medicare Advantage Beneficiaries
Source: JAMA Health Forum. 2023 Aug 18;4(8):e232517. doi: 10.1001/jamahealthforum.2023.2517 (PMC10439482; doi:10.1001/jamahealthforum.2023.2517)
Supplement: Supplement 1. — eMethods. STROBE Compliance eFigure 1. Dual-Eligible Postacute Care Service Use by NHATS Survey Round and Managed Care Enrollment, 2015-2017 eTable 1. Self-Reported Use of Postacute Care by NHATS Survey Round and Managed Care Enrollment for Subgroups eTable 2. Self-Reported Outcomes by Managed Care Enrollment for Subgroups eTable 3. NHATS Survey Questions Related to Postacute Care eFigure 2. CONSORT Diagram for Study Population [file jamahealthforum-e232517-s001.pdf]

Supplemental Online Content

Achola EM, Stevenson DG, Keohane LM. Postacute care services use and outcomes among traditional Medicare and Medicare Advantage beneficiaries. *JAMA Health Forum*. 2023;4(8):e232517. doi:10.1001/jamahealthforum.2023.2517

eMethods. STROBE Compliance

eFigure 1. Dual-Eligible Postacute Care Service Use by NHATS Survey Round and Managed Care Enrollment, 2015-2017

eTable 1. Self-Reported Use of Postacute Care by NHATS Survey Round and Managed Care Enrollment for Subgroups

eTable 2. Self-Reported Outcomes by Managed Care Enrollment for Subgroups

eTable 3. NHATS Survey Questions Related to Postacute Care

eFigure 2. CONSORT Diagram for Study Population

This supplemental material has been provided by the authors to give readers additional information about their work.

© 2023 Achola EM et al. *JAMA Health Forum.*

eMethods. STROBE Compliance

The study complies with the main requirements in STROBE guidelines for a cohort study: title and abstract, introduction, methods, results, discussion, and other information. The background, study objectives, and study design are clearly included in the main text. Data sources, study size (see eFigure2 for more information), and statistical methods are also outlined. Additionally, results, interpretation, and limitations are in the discussion section. Lastly, funding information for the study is included.

© 2023 Achola EM et al. *JAMA Health Forum.*

eFigure 1. Dual-Eligible Postacute Care Service Use by NHATS Survey Round and Managed Care Enrollment, 2015-2017

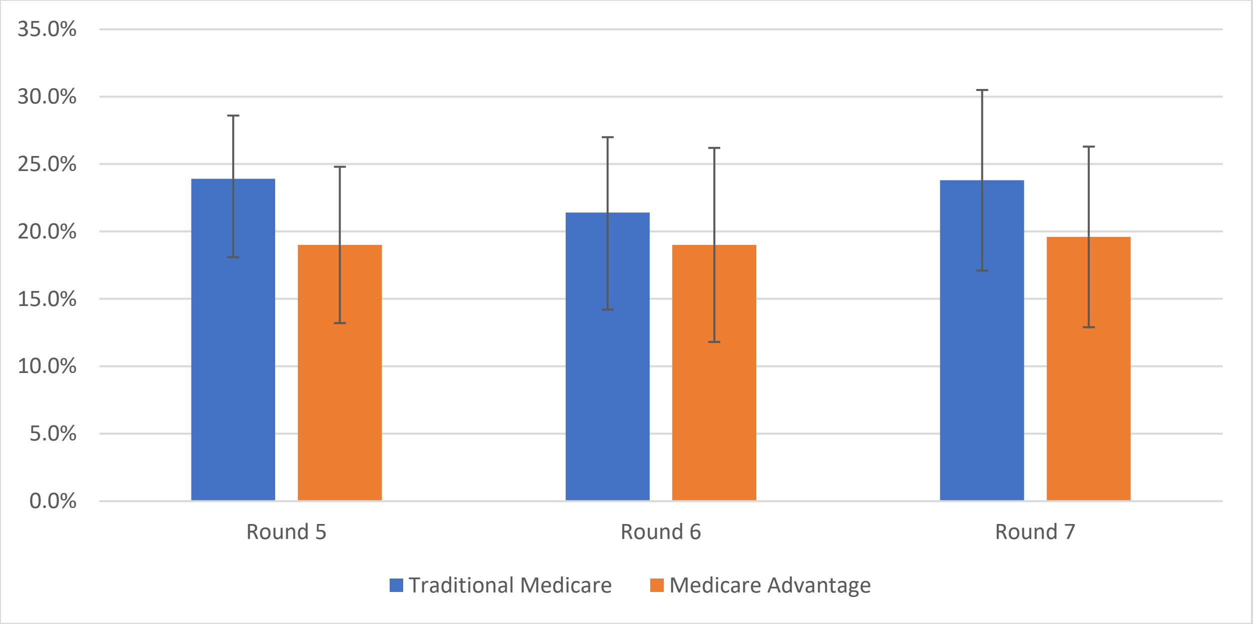

Note: Authors' analysis of National Health and Aging Trends Survey (NHATS) data linked to Medicare enrollment data, years 2015-2017. Post-acute care (PAC). Percentages were weighted to account for the complex survey design. Comparisons between groups based on Pearson's chi-squared tests, results significant at 5% level. *p-value <0.05, ** <0.01, *** <0.001

© 2023 Achola EM et al. *JAMA Health Forum.*

eTable 1. Self-Reported Use of Postacute Care by NHATS Survey Round and Managed Care Enrollment for Subgroups

	Round 5	Round 6	Round 7
**Fall in the last year (%)**			
Traditional Medicare	33.1 (30.5, 35.9) **	30.7 (28.1, 33.4) *	32.4 (28.7, 36.3) *
Medicare Advantage	25.6 (22.3, 29.3)	25.7 (21.9, 29.9)	26.0 (21.2, 31.3)
**Hip or knee surgery in the last year (%)**			
Traditional Medicare	75.4 (66.6, 82.5)	78.5 (70.2, 85.0)*	75.8 (67.1, 82.8)
Medicare Advantage	83.6 (73.6, 90.3)	61.6 (45.3, 75.6)	66.8 (53.9, 77.5)
**History of arthritis or osteoporosis (%)**			
Traditional Medicare	26.5 (24.4, 28.7) ***	26.5 (24.4, 28.6) **	28.5 (25.8, 31.4) ***
Medicare Advantage	19.2 (16.7, 21.9)	20.3 (17.1, 23.9)	20.3 (17.3, 23.8)

Note: Authors' analysis of National Health and Aging Trends Survey (NHATS) data linked to Medicare enrollment data, years 2015-2017. Percentages were weighted to account for the complex survey design. *p-value <0.05, ** <0.01, *** <0.001

© 2023 Achola EM et al. *JAMA Health Forum.*

eTable 2. Self-Reported Outcomes by Managed Care Enrollment for Subgroups

	**Fall in the last year (n = 1,184)**		**Hip or knee surgery in the last year (n = 283)**		**History of arthritis or osteoporosis (n = 1,902)**	
	Traditional Medicare (n = 785)	Medicare Advantage (n = 399)	Traditional Medicare (n = 181)	Medicare Advantage (n = 102)	Traditional Medicare (n = 1,256)	Medicare Advantage (n = 646)
**Functioning improved in post-acute services (%)**						
No improvement or worsened	29.3 (25.6, 33.2)	39.9 (35.2, 44.9)**	11.7 (7.0, 18.9)	24.0 (14.3, 37.2)*	28.6 (25.6, 31.8)	38.3 (34.2, 42.6)***
Improvement	70.7 (66.8, 74.4)	60.1 (55.1, 64.8)	88.3 (81.1, 93.0)	76.0 (62.8, 85.7)	71.4 (68.2, 74.4)	61.7 (57.4, 65.8)
**Currently receiving post-acute services (%)**						
No	76.7 (73.2, 79.8)	75.8 (70.6, 80.3)	75.4 (66.5, 82.5)	78.3 (65.5, 87.3)	77.8 (74.9, 80.5)	76.2 (72.6, 79.5)
Yes	23.3 (20.2, 26.8)	24.2 (19.7, 29.4)	24.6 (17.5, 33.5)	21.7 (12.7, 34.5)	22.2 (19.5, 25.1)	23.8 (20.5, 27.4)
**Met goals when services ended (%)**						
No	25.3 (21.4, 29.8)	29.3 (23.1, 36.4)	15.4 (9.6, 23.9)	20.6 (13.6, 29.9)	24.6 (21.3, 28.3)	29.4 (24.5, 34.9)
Yes	74.7 (70.2, 78.6)	70.7 (63.6, 76.9)	84.6 (76.1, 90.4)	79.4 (70.1, 86.4)	75.4 (71.7, 78.7)	70.6 (65.1, 75.5)
**Functioning improved when services ended (%)**						
No improvement or worsened	57.5 (52.8, 62.1)	60.0 (52.7, 67.0)	33.0 (23.9, 43.5)	34.1 (21.9, 48.8)	54.2 (50.0, 58.4)	56.6 (50.8, 62.1)
Improvement	42.5 (37.9, 47.2)	40.0 (33.0, 47.3)	67.0 (56.5, 76.1)	65.9 (51.2, 78.1)	45.8 (41.6, 50.0)	43.4 (37.9, 49.2)

Note: Authors' analysis of National Health and Aging Trends Survey (NHATS) data linked to Medicare enrollment data, years 2015-2017.Percentages were weighted to account for the complex survey design. Comparisons between groups based on Pearson's chi-squared tests, results significant at 5% level. *p-value <0.05, ** <0.01, *** <0.001

© 2023 Achola EM et al. *JAMA Health Forum.*

eTable 3. NHATS Survey Questions Related to Postacute Care

Survey variable	Survey question
Months of rehab	In the last year, for about how many months altogether did {you/SP} receive rehab services?
Weeks of rehab	In the last year, for about how many weeks altogether did {you/SP} receive rehab services?
Rehab post-surgery	Did {you/SP} receive rehab to help {you/him/her} recover following surgery?
Main medical condition for surgery	Please look at this card and tell me, what was the main medical condition for which {you/SP} had surgery?
Main medical condition for rehab	Please look at this card and tell me, what was the main medical condition for which {you/SP} received rehab in the last year?
Receive rehab overnight hospital, NH, rehab	In the last year, did {you/SP} receive rehab as an overnight patient in a hospital, nursing home, or rehab facility?
Receive rehab outpatient	In the last year, did {you/SP} receive rehab at an outpatient, clinic, or doctor's office or therapist's office?
Receive rehab home	In the last year, did {you/SP} receive rehab at home?
Receive rehab somewhere else [a]	In the last year, did {you/SP} receive rehab somewhere else?
Functioning improved in rehab	While {you were/SP was} receiving rehab services in the last year, did {your/his/her} functioning and ability to do activities improve, get worse, or stay about the same?
Still receiving rehab	{Are you/Is SP} still receiving rehab services?
Met goals when rehab ended	When {your/SP's} rehab services ended, had {you/SP} met all or most of {your/his/her} goals?
Functioning improved when rehab ended	Since {your/SP's} services ended, have {you/his/her} functioning and ability to do activities improved, got worse, or stayed the same?

Note: [a] Responses included: as an overnight patient in a hospital, nursing home, or rehab facility; outpatient, clinic, or doctor's office or therapist's office; home; and somewhere else(unspecified)

© 2023 Achola EM et al. *JAMA Health Forum.*

eFigure 2. CONSORT Diagram for Study Population

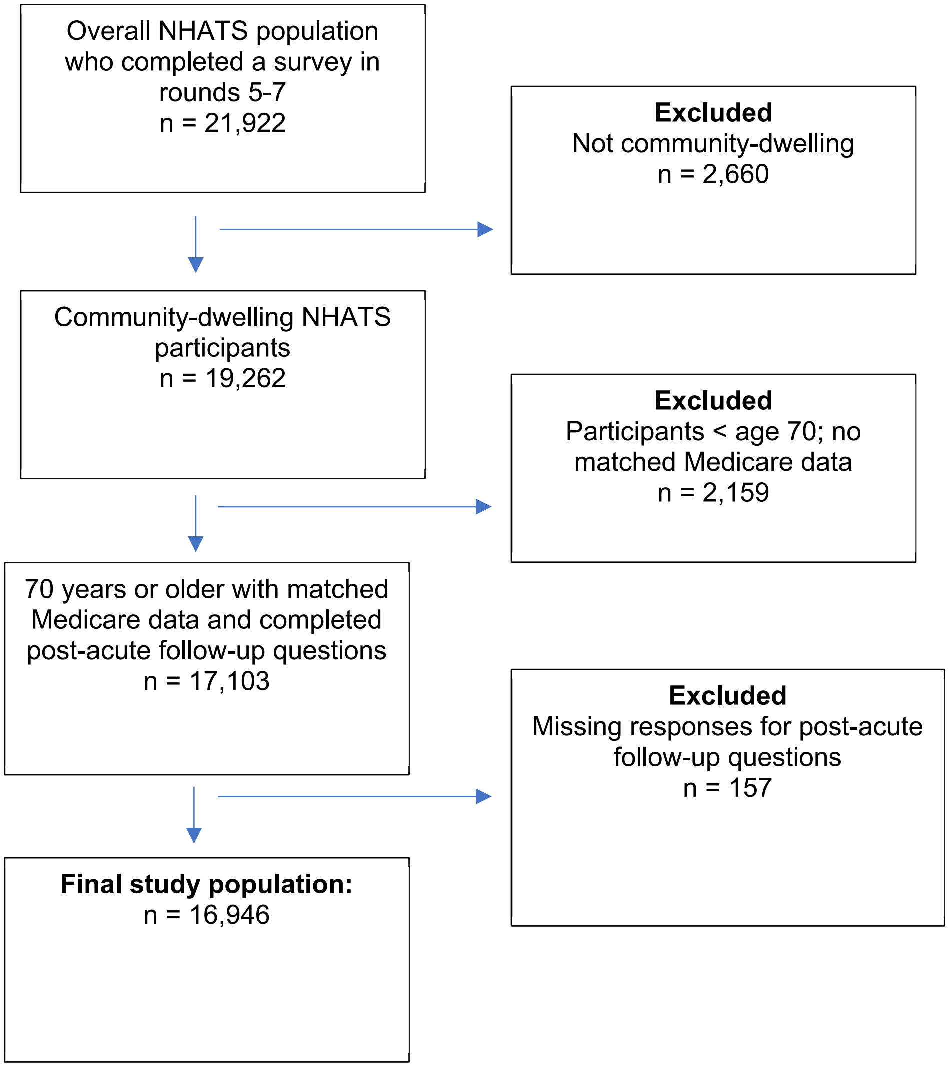

© 2023 Achola EM et al. *JAMA Health Forum.*
