# Supplementary material for: Postacute Care Services Use and Outcomes Among Traditional Medicare and Medicare Advantage Beneficiaries
Source: JAMA Health Forum. 2023 Aug 18;4(8):e232517. doi: 10.1001/jamahealthforum.2023.2517 (PMC10439482; doi:10.1001/jamahealthforum.2023.2517)
Supplement: Supplement 2. — Data Sharing Statement [file jamahealthforum-e232517-s002.pdf]

Data Sharing Statement

Achola. Postacute Care Services Use and Outcomes Among Traditional Medicare and Medicare Advantage Beneficiaries. *JAMA Health Forum*. Published August 18, 2023. doi:10.1001/jamahealthforum.2023.2517

Data
Data available: No
